# International survey in four European countries on diagnostic reference levels based on clinical indications in computed tomography

**DOI:** 10.1186/s13244-026-02241-4

**Published:** 2026-03-12

**Authors:** Alexander A. Schegerer, Kerstin Jungnickel, Michael Walz, Michael Verius, Julian Singer, Bernhard Renger, Nadia Oberhofer, Constance Müller, Roman Menz, Bärbel Madsack, Martin Fiebich, Rainer Eßeling, Markus Borowski, Babak Bazrafshan, Josefin Ammon, Christoph Aberle, Georg Stamm

**Affiliations:** 1 Department of Radiation Protection and Radiological Processes, Hirslanden Hospitals, Opfikon-Glattpark, Switzerland; 2 Institute of Diagnostic and Interventional Radiology, Municipal Hospital Magdeburg, Magdeburg, Germany; 3 Medical Office for Quality Assurance in Radiology, Nuclear Medicine and Radiation Therapy (Ärztliche Stelle Hessen), TÜV SÜD Life Service GmbH, Frankfurt, Germany; 4 Department of Radiology, Medical University Innsbruck, Innsbruck, Austria; 5 MPS Medical Physics and Radiation Protection GmbH, Schwabach, Germany; 6 Department of Diagnostic and Interventional Radiology, School of Medicine & Health, Klinikum Rechts der Isar, Technical University of Munich, Munich, Germany; 7 Azienda Sanitaria dell’Alto Adige, Serviczio Aziendale di Fisica Sanitaria, Bolzano, Italy; 8 Clinic for Radiology, University Hospital Münster, Münster, Germany; 9 Department of Radiology, University Hospital Basel, University of Basel, Basel, Switzerland; 10 Institute of Medical Physics and Radiation Protection, University of Applied Sciences, Giessen, Germany; 11 Institute of Radiology and Nuclear Medicine, Municipal Hospital Braunschweig, Braunschweig, Germany; 12 Isotopenlabor & Strahlenschutz, Universitätsklinikum Tübingen, Tübingen, Germany; 13 Institute of Medical Physics, Nuremberg General Hospital, Paracelsus Medical University, Nuremberg, Germany; 14 Institute for Diagnostic and Interventional Radiology, University Medical Center Göttingen, Göttingen, Germany

**Keywords:** Computed tomography, Radiation dose, Diagnostic reference levels, Radiation protection, Europe

## Abstract

**Objective:**

To collect and analyze radiation dose metrics from 23 commonly performed CT procedures, for which no national DRLs have yet been defined, as part of an international cooperation. The body region, the clinical task, and the relevant technical parameters were considered to define clinical diagnostic reference levels (DRLs).

**Materials and methods:**

During 2022–2023, a multicenter study was performed in Austria, Germany, Italy, and Switzerland. Healthcare providers supplied processed data from their dose management systems. The 5%-level was used to assess the statistical significance of dose differences between the countries of participants, as well as age and manufacturer of the scanners.

**Results:**

Dose metrics from 87 CT systems in academic and non-academic, public and private healthcare facilities were analyzed. Dose values from the same body region but for different clinical tasks and/or using different techniques might differ significantly. For the same procedures, median dose values varied between systems by a factor of up to 39. Approximately a third of the surveyed systems showed doses within the DRLs suggested in this study, while 6% do not fulfill any DRL. No significant dose differences were observed between systems when comparing age, manufacturer or country.

**Conclusions:**

In this international study, clinical DRLs were suggested for the first time for many different CT procedures, including dose-intensive procedures such as cerebral perfusion or the CT-guided interventional periradicular therapy. The diversity in protocol optimization is one of the main reasons for dose variations. Establishing the proposed DRLs can help harmonize exposure practice across country borders.

**Critical relevance statement:**

In this international multicenter study, clinical task-based DRLs were suggested for 23 CT procedures to promote the optimization of the exposure practice, to reduce dose variations among institutions, even across national borders and to strengthen international cooperation among users.

**Key Points:**

- Analysis and differences in exposure practices in 23 different CT examinations in four neighboring European countries.
- Dose values for the same clinical tasks vary considerably between facilities using the same technology, but on average, not between countries.
- Establishing diagnostic reference levels helps to harmonize the exposure practice across country borders for frequently performed procedures.

**Graphical Abstract:**

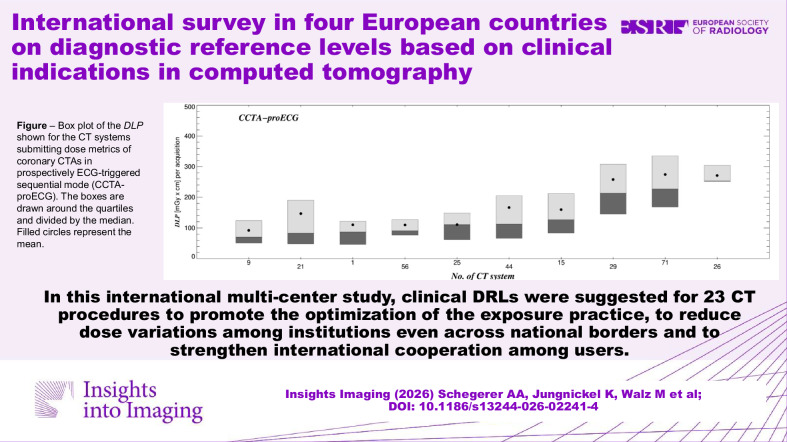

## Introduction

International recommendations [1, 2] and national laws (e.g., [3, 4]) require users of ionizing radiation in medicine to optimize radiological procedures (known as the ALARA principle, “as low as reasonably achievable”). However, implementing the ALARA principle in everyday clinical practice can be difficult, as various factors must be considered, such as the technical equipment and the minimum image quality required for diagnosis.

An important step in the evaluation of optimization is the use of diagnostic reference levels (DRLs), which are compared with local dose-relevant parameters. According to the International Commission on Radiological Protection, users should investigate the causes for exceeding the DRLs. If the excess is unjustified, measures should be taken to achieve compliance [5]. Numerically, a DRL is defined as the 3rd quartile of the distribution of median values of a dose quantity (e.g., dose length product, DLP, or computed tomography dose index, CTDI_vol_) acquired at different X-ray systems.

The common approach in recent years was to define DRLs for selected body regions (anatomical DRLs, e.g., DRL for brain) [6]. However, for better efficiency, it was recommended to additionally take into account the clinical task (e.g., the differentiation of pathological from healthy brain areas after vascular occlusion) and the procedural technique (e.g., perfusion measurements with toggling table) [5, 7]. Based on this CAP approach (clinical task, anatomical region, procedural technique), the term clinical DRL was introduced, which has already been defined for procedures in CT [7, 8], interventional radiology (IR) [9, 10] and radiography [11–13].

DRLs are usually set by authorities for a small number (5–20 per modality, [7]) of frequently performed and/or dose-intensive procedures. If national DRLs are missing, users might rely on dose parameters of comparable other protocols, DRLs from other countries, or those of internationally active expert groups. The concern that technical equipment and exposure practice differ between facilities in different countries and that a comparison of dose-related parameters is therefore inappropriate has not proven to be true (at least for countries participating in a recent study [12]).

The German Roentgen Society’s working group of physics and technology (APT) launched a survey to acquire dose-related data and to define DRLs for frequently performed procedures without national DRLs. APT members and other well-known colleagues from practices and clinics were asked to take part in the survey. In order to get a larger database, various facilities in Austria, Germany, Italy, and Switzerland were contacted. The aim of this paper is to report on the survey on CT procedures and to propose clinical DRLs.

## Materials and methods

Anonymized and aggregated dose metrics were analyzed in this study. All institutional review boards confirmed that no national regulations and international declarations for the protection of human subjects were affected.

Dose indices from 18 diagnostic and 1 CT-guided interventional procedures were selected. For establishing clinical DRLs, it is common practice to use the CAP approach for defining the clinical task, anatomical location, and procedural technique (Table 1). Additionally, DRLs were applied for 4 auxiliary procedures (3 localizers and the premonitoring acquisition to determine the ROI for the monitoring to detect the arrival of the contrast bolus). In total, 23 “reference procedures” were surveyed. The monitoring scans have a very wide spread of CTDI_Vol_, some significant above 100 mGy, were not included in the study.

**Table 1:** Name, clinical task, anatomical location (scanning area), and, where applicable, procedural technique and/or phase of the reference procedures surveyed in this international multicenter study

		Clinical task	Anatomical location	Technique (phase)
1	CTP	Cerebral vascular occlusion in stroke, differentiation of salvageable ischemic brain tissue from the irrevocably damaged infarcted brain	Two brain segments	Perfusion measurement with «toggling table»
2	CT petrous bone	Fracture, malformation or calcification of the inner ear	Tegmen tympani—mastoid process	Native
3	CTA-LE	Pulmonary artery embolism	Aortic arch—just above the diaphragm	CTA
4	Calcium scoring	Determination of calcification and craniocaudal extent of the heart	Aortic bulb—cardiac apex	
5	CCTA-retroECG			retroECG
6	CCTA-proECG	Disease of the heart and coronary vessels	Aortic bulb (possibly with aortic arch)—cardiac apex	proECG
7	CCTA-HPH			HPH
8	preTAVI/R-heart	Preprocedural CT imaging before TAVI/TAVR (preTAVI): examination of the anatomy of the aortic valve and of the vascular system	Aortic bulb—sino-tubular junction	proECG
9	preTAVI/R-aorta		Aortic bulb—femoral arteries beyond the femoral head	CTA (non-ECG-synchronized)
10	preTAVI/R-heart & aorta		Aortic bulb—femoral arteries beyond the femoral head	HPH
11	CT stone	Urolithiasis (CT stone)	Upper kidney pole—ischium	Native
12	PRT	Local injection of anti-inflammatory medication at the nerve root	One to two disc spaces	CT-guided intervention (could be combined with diagnostic CT scans of the spine)
13	CT-shoulder		Shoulder joint and the proximal and distal parts of the adjacent bones	
14	CT-elbow		Elbow joint and the proximal and distal parts of the adjacent bones	
15	CT-wrist		Wrist joint and the proximal and distal parts of the adjacent bones	
16	CT-knee	Complex joint pathology	Knee joint and the proximal and distal parts of the adjacent bones	Positioning of the joint within the gantry must be considered
17	CT-upper ankle		Upper ankle joint and the proximal and distal parts of the adjacent bones	
18	CT-foot		Foot and the distal part of the tibia and fibula	
19	Multiple myeloma	Bone lesions as a result of multiple myeloma	Whole body	Native
20*	localizer,-trunc PA/AP	Auxiliary acquisitions to define the area to be scanned; also known as scout, topogram or surview	Body boundaries (neck, trunk or extremities) adapted to the clinical task	PA/AP view
21*	localizer,-trunc LAT		Body boundaries (neck, trunk or extremities) adapted to the clinical task (outside the head)	LAT view
22*	localizer-head LAT		Head	LAT view
23*	Premonitoring	Auxiliary acquisition to define the location for monitoring the contrast agent bolus	Small segment of the body trunk (scan length is a slice with a detector pixel width)	Small slice is sufficient

Depending on the scanner and patient, one of the acquisitions 5, 6 or 7 is performed. Acquisition 9 follows acquisition 8; acquisition 10 is an alternative to acquisitions 8 and 9. Procedures marked with an asterisk (no. 20–23) are auxiliary acquisitions

*CT* computed tomography, *CTP* CT perfusion, *CTA* CT angiography, *LE* lung embolism, *CCTA* coronary CTA, *retroECG* retrospectively ECG-gated mode, *proECG* prospectively ECG-triggered sequential mode (step-and-shoot), *HPH* high-pitch helical mode, *preTAVI/R* preprocedural CT imaging before transcatheter aortic valve implantation/replacement, *PRT* CT-guided periradicular therapy, *AP* anterior-posterior, *PA* posterior-anterior, *LAT* lateral

Experiences from previous surveys served as guidelines concerning format, content and the execution of this survey [7, 14–16]. To minimize the effort, the processed output of dose management systems (DMS) was used for this study. A correct functioning of the DMS [17] was ensured by the physicists who were responsible for the data included in this study. The following information was requested:

1. Key data of the surveyed CT systems.
2. Filter criteria used in the DMS for the assignment (“mapping”) of CT acquisitions to the reference procedures.
3. For 18 procedures (Table 1), the quartiles and mean of the CTDI_vol_ and DLP, and—if available—the median value of the tube voltage (*U*) computed with the DMS from at least 10 CT acquisitions (within 1 year) of adult patients (age > 16 years old).
4. For the 4 auxiliary scans, the quartiles and mean of the CTDI_vol_.
5. For the CT-guided periradicular therapy (PRT), the sum of the DLPs of all acquisitions of a procedure (excluding the localizers).

For all participating facilities, the included CT systems are subject to periodic maintenance and quality assurance supervised by local authorities. In accordance with the international standard [18], the deviation of the displayed dose from measurements must be less than 20%. All participants reported compliance with national DRLs and sought to investigate reasons for any excess dose. They were experienced professionals in dose optimization with more than 10 years of practice in this field.

The survey was conducted in 2022–2023. A detailed review process ensured that all collected data were in the correct format, order of magnitude, and properly mapped to one of the reference procedures. In case of doubt regarding potentially incorrect information (e.g., particularly high CTDI_vol_), web conferences among participants were organized to verify, correct, or complete the data and solve any remaining issues. Analysis of all data and assessment of the clinical DRLs were made. All contributing facilities received a comparison of their local exposure levels to the DRLs defined in this study.

The statistical evaluation included the calculation of the quartiles and mean from the distribution of the reported median values of DLP, CTDI_vol_ and *U* for each reference procedure. As is commonly accepted, the 3rd quartile of median values was defined as DRL. Depending on how many subgroups (e.g., country, year of installation, manufacturer) had to be considered, either the Mann–Whitney or the Kruskal–Wallis test was used for assessing the statistical significance of dose differences between different procedures or different subgroups. For all statistical tests performed with the program IDL (version 8.7.2., Harris Geospatial Solutions), a significance level of *p* < 0.05 was used.

## Results

An international network of medical physicists, radiographers, and radiologists was established. Dose-related data were obtained from 2 Austrian (despite contacting multiple institutions in Austria), 54 German, 10 Italian, and 21 Swiss CT systems in academic, non-academic, public, and private healthcare facilities. On average, the systems were 7.8 years old in 2023, and 75% of the systems were products of one manufacturer. No significant dose differences were found between CT scanners of different ages (older or younger than the average age) and between four CT manufacturers.

**Fig. 1:**
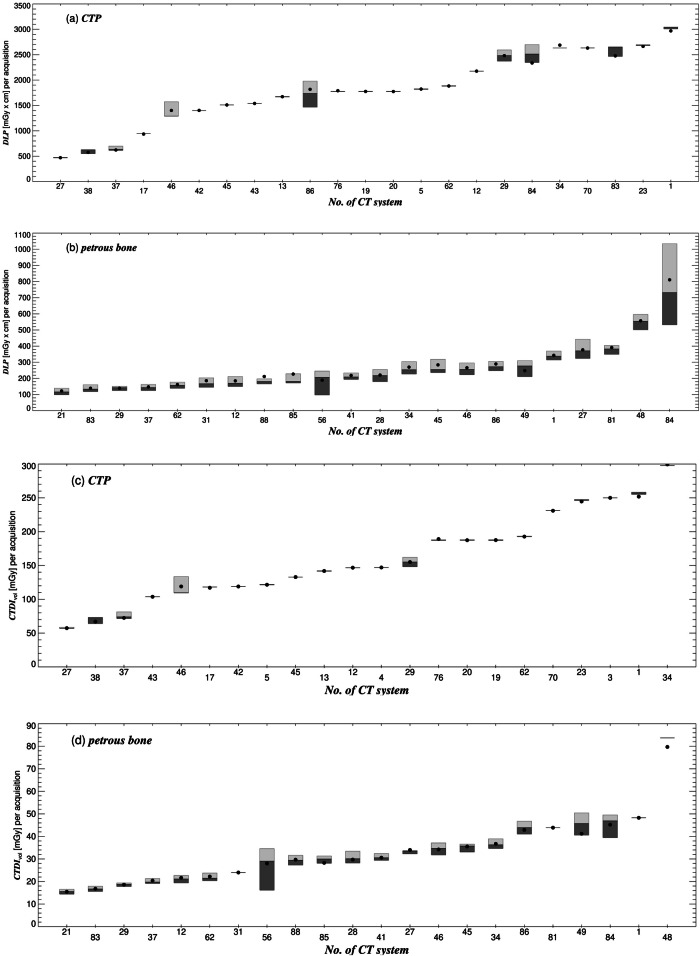
Box plots of the dose length product (DLP) and pitch-corrected computed tomography dose index (CTDI_vol_) per acquisition shown for the CT systems (*x*-axis) that submitted dose-related parameters for (**a**, **c**) CTP and (**b**, **d**) the CT exam of the petrous bone. The boxes are drawn around the 1st and 2nd (dark gray boxes) as well as 2nd and 3rd quartiles (light gray) and divided by the median (2nd quartile). Filled circles represent the mean

**Fig. 2:**
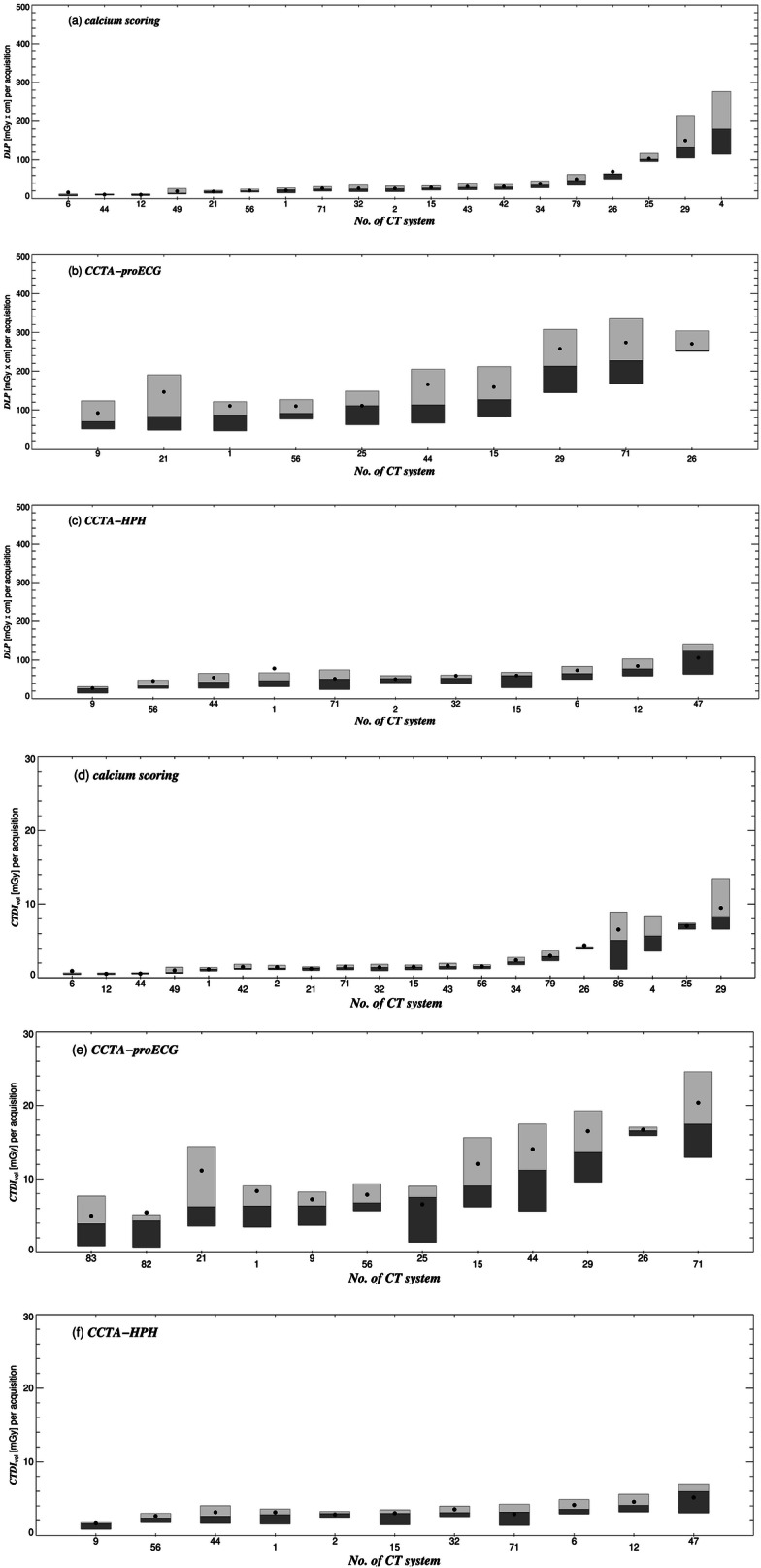
Box plots of the DLP and CTDI_vol_, shown for the CT systems that submitted dose-related data for calcium scoring (**a**, **d**), coronary CT angiography (CTA) in prospectively ECG-triggered sequential mode (**b**, **e**) and CTA in high-pitch helical mode (**c**, **f**). The boxes are drawn around the 1st and 2nd (dark gray boxes) as well as the 2nd and 3rd quartile (light gray) and divided by the median (2nd quartile). Filled circles represent the mean

As the facilities involved were specialized in different medical fields, they could not submit dose-related parameters equally for all the reference procedures. The number of systems that provided data for the corresponding procedures, the quartiles, as well as the means of the distribution of median values are listed in Table 2. As examples, Figs. 1, 2 show the dose data from each contributing system for cerebral CT perfusion (CTP), CT petrous bone, calcium scoring, coronary CTA in prospectively ECG-triggered mode (CCTA-proECG, step-and-shoot mode), and CCTA in high-pitch helical mode (CCTA-HPH).

**Table 2:** Number of contributing CT systems, 1st, 2nd and 3rd quartile as well as mean of median values of the distribution of DLP and CTDI_vol_ and 2nd quartile of the distribution of median values of the tube voltage for the 23 surveyed reference procedures

	Reference procedures	Number of contributing CT systems	1st quartile		2nd quartile		3rd quartile (DRL)		Mean		2nd quartile
			DLP (mGy·cm)	CTDI_vol_ (mGy)	DLP (mGy·cm)	CTDI_vol_ (mGy)	DLP (mGy·cm)	CTDI_vol_ (mGy)	DLP (mGy·cm)	CTDI_vol_ (mGy)	*U* (kV)
1	CTP	22	1430	120	1780	150	**2510**	**190**	1810	160	80
2	CT petrous bone	22	170	22	210	30	**280**	**44**	260	34	120
3	CTA-LE	52	130	3.6	170	5.0	**220**	**6.4**	190	5.7	100
4	Calcium scoring	20	19	1.2	25	1.4	**43**	**3.5**	43	3.5	110
5	CCTA-retroECG	2	450	30	720	48	**1000**	**65**	720	48	95
6	CCTA-proECG	12	87	6.3	110	7.1	**210**	**12**	140	9.1	100
7	CCTA-HPH	11	43	2.6	51	3.0	**63**	**3.4**	57	3.2	95
8	preTAVI/R-heart	7	150	13	270	22	**480**	**31**	310	21	80
9	preTAVI/R-aorta	7	210	3.2	310	4.8	**330**	**5.0**	280	4.3	100
10	preTAVI/R-heart & aorta	4	160	3.3	270	4.5	**390**	**5.3**	280	4.3	100
11	CT stone	37	150	3.8	180	4.4	**349**	**5.1**	190	4.6	120
12	PRT	15	60		140		**260**		470		120
13	CT-shoulder	43	160	8.7	200	10	**270**	**13**	230	12	110
14	CT-elbow	28	88	5.1	99	6.8	**150**	**11**	130	8.5	120
15	CT-wrist	39	65	4.9	92	6.7	**130**	**11**	100	7.6	120
16	CT-knee	41	91	3.8	120	5.3	**160**	**7.7**	150	6.2	120
17	CT-upper ankle	33	76	4.0	98	4.8	**150**	**7.1**	110	5.9	120
18	CT-foot	30	79	4.1	98	6.1	**180**	**9.2**	130	6.9	120
19	Plasmacytoma	18	390	2.2	581	3.5	**810**	**5.5**	680	3.7	120
20	localizer-trunc PA/AP	40	-	0.01	-	0.03	**-**	**0.09**	-	0.06	100
21	localizer-trunc LAT.	24	-	0.06	-	0.08	**-**	**0.1**	-	0.1	120
22	localizer-head LAT.	12	-	0.1	-	0.2	**-**	**0.2**	-	0.2	140
23	Premonitoring	12	-	0.9	-	1.1	**-**	**1.9**	-	1.4	115

The 3rd quartiles represent the corresponding DRLs (bold font). For PRT, the specified mean value and quartiles are computed from the distribution of median values of the total DLPs, which are summed over all individual acquisitions of a procedure. The dose indices for the reference procedures 1, 2 and 22 refer to the head CT dosimetry phantom (16 cm diameter). The dose indices for the remaining reference procedures refer to the body CT dosimetry phantom (32 cm diameter)

*CT* computed tomography, *DLP* dose length product, *CTDI*_vol_ pitch-corrected CT dose index, *U* voltage, *CTP* cerebral CT perfusion, *CTA* CT angiography, *LE* lung embolism, *CCTA* coronary CTA, *retroECG* retrospectively ECG-gated mode, *proECG* prospectively ECG-triggered sequential mode, *HPH* high-pitch helical mode, *preTAVI/R* preprocedural CT imaging before transcatheter aortic valve implantation/replacement, *PRT* CT-guided periradicular therapy, *AP* anterior-posterior, *PA* posterior-anterior, *LAT* lateral

The ratio of the 3rd and 1st quartile of the dose distribution (inter-quartile ratio) is a measure of the intra-system dose variation. It shows how strongly the dose values are spread for the same reference procedure and the same system. For the same procedure/protocol, dose variations within a system should be caused by the system’s automatic exposure control for different patient thicknesses. In the case of CTDI_vol_, particularly high intra-system discrepancies were found for CCTA-proECG (8.2 at maximum) and calcium scoring (7.6), although in the latter procedure, the discrepancy is only present in three systems (Fig. 2). The median intra-system dose variation is 1 for CTP, as fixed parameter values were used. Averaged over all systems and reference procedures, the intra-system dose discrepancy is 3 for CTDI_vol_ and DLP.

For the same reference procedure, the inter-system dose variation can be large, i.e., the median values of DLP and CTDI_vol_ of the surveyed systems can also greatly differ. The median values of the DLP differ by a maximum factor of 36 for plasmacytoma and 39 for PRT between the surveyed scanners (for PRT, the DLP indicated is the sum of DLPs over all individual acquisitions).

The number of systems using optimized dose protocols for all reference procedures of this study, i.e., consistently complying with the proposed DRLs for DLP and CTDI_vol_, is 42% and 32%, respectively. In contrast, 7% and 5% of the systems do not comply with any of these DRLs.

The 2nd quartile of the median values of the tube voltage is shown in Table 2. The values correspond largely to the national recommendation [19]. While the tube voltage of acquisitions with contrast agent, e.g., CT angiographies (CTA), is 100 kV or less, a voltage of 120 kV is selected for acquisitions without contrast agent.

In CCTA, there are significant differences in CTDI_vol_ between retrospectively ECG-gated CTA (CCTA-retroECG), CCTA-proECG, CCTA-HPH and calcium scoring. Between CT-wrist and CT-elbow or between CT-upper ankle and CT-foot, there were no statistically significant dose differences.

The German and the Swiss systems do not show any significant dose differences for the reference procedures. The number of Austrian and Italian facilities contributing to each reference procedure is too small to be considered for statistical significance. However, the level of their dose indices is mostly in good agreement with data from German and Swiss facilities.

## Discussion

With the help of DRLs, the exposure practice can be assessed and harmonized by identifying procedures that need optimization [7]. Within the same body region, there could be different clinical tasks and/or different procedural techniques, which may be associated with significantly different patient doses. In this case, different DRLs should be used [5]. For instance, to rule out coronary heart disease, CCTA-retroECG, CCTA-proECG or CCTA-HPH is performed depending on the available techniques and the patient’s condition, e.g., whether cardiac arrhythmia is present. Compared to CCTA-retroECG, the DRLs for DLP decrease significantly by 65% and 90% for CCTA-proECG and CCTA-HPH, respectively. Contrarily, the dose values among the procedures of the upper extremities and among the procedures of the lower extremities do not significantly differ. For optimization reasons, it would therefore be sufficient to define a common DRL for the examinations of the elbow and wrist and a common DRL for the examinations of the knee, upper ankle and foot.

This study emphasizes the need to establish clinical DRLs for more regularly performed CT procedures without existing national DRLs. It was found that there can be large dose differences between the systems, in particular for the CT intervention PRT, which correspond to the outcome of earlier studies on IR (e.g., [10]). Approximately, two-thirds of the surveyed systems exceed at least the DRL of one of the reference procedures, 7% (6 systems, 4 of them are older systems with an 8- or 16-row detector, 2 are more modern systems with a 125- and 192-detector) do not meet any new DRL. The reason for these dose variations may be the lack of knowledge about the doses expected under good practice and/or the lower attention paid to procedures for which DRLs have not yet been stated. It cannot be completely dismissed, however, whether the scanner type also influences the dose. Nevertheless, the current survey shows potential for dose reduction in many of the included CT systems through the optimization of examination protocols.

The CTDI_vol_ listed in the DICOM-structured dose report of CTPs is the sum of the CTDI_vol_ from rotations at different holding positions of the toggling table in the skull area. The total radiation exposure during CTP is affected by various scan parameters, such as the CTDI_vol_ per rotation, the number and time intervals between the rotations and the scan length. The suggested DRLs for CTP correspond to a previous study [20] and are well below the critical value for deterministic effects [21]. In most participating systems, there is no intra-system dose variation (Fig. 1a, c) since most participating facilities always perform CTP with fixed scan parameters. The aim is to achieve a minimum coverage of the perfusion of the contrast medium in the patient’s skull to meet the specifications of the evaluation software [22, 23]. In a previous study, it was shown that sufficient coverage could be achieved on various scanners with CTDI_vol_ values ranging from 110 to 130 mGy, a tube voltage of 70–80 kV, 24 individual rotations and a total measurement time of 45 s (with a high scan rate of 18 × 1.5 s during arterial phase and a low scan rate of 6 × 3 s during venous phase) [24].

The different procedural techniques used for CCTA are described in [25]. In this study, dose-intensive CCTAs-retroECG were used sporadically, at two CT systems only. If the heart rate is low (e.g., < 65 bpm) and there is no cardiac arrhythmia, CCTAs-proECG or even CCTAs-HPH, if available, should be used instead [26, 27]. Image contrast can be increased (and radiation exposure decreased) if the tube voltage is lowered below a value of 120 kV, preferably to 100 kV for patients with a BMI < 30 kg/m^2^. In this study, the median value of the tube voltage was 100 kV. With some modern scanners, the tube voltage can be lowered to 70 kV (preferably for patients with a chest diameter of < 32 cm, [28]), which results in significant dose saving: a DLP of 40–50 mGy·cm corresponds to an effective dose in the sub-millisievert range [29].

In our study, there were only a few participating facilities that regularly perform preprocedural CT imaging prior to transcatheter aortic valve implantation/replacement (preTAVI/preTAVR). The DRLs reported for these procedures are still subject to greater uncertainty than for the other reference procedures. Usually, a CCTA-proECG scan (preTAVI/R-heart) is combined with a CTA of the aorta (preTAVI/R-aorta). Alternatively, the heart and aorta can be scanned in HPH mode in one acquisition (preTAVI/R-heart & aorta). The dose-related parameter values determined here were similar to or lower than the values that were found in previous studies [30, 31]. The DLP for preTAVI/R-heart & aorta is reduced by more than half compared to the combined procedure. There is also a considerable need for dose optimization, as the CTDI_vol_ values are 56% and 160% higher for preTAVI/R-heart and preTAVI/R-heart & aorta than for CCTA-proECG and CCTA-HPH, respectively.

In PRT, there were major differences between the participating facilities in terms of the number of acquisitions and the dose-relevant parameters. In some facilities, PRT starts with an overview scan of the lumbar spine, followed by CT-fluoroscopy, in which the drug is injected, performed in helical or sequential CT-mode. In sequential mode, each acquisition can consist of a different number of rotations, depending on the radiologist’s approach. The dose-relevant parameter values add up accordingly. In some cases, the PRT was completed with a control scan to assess the distribution of the drug in the disc spaces. Due to this heterogeneous approach, no DRL was proposed for the acquisition. Instead, it was considered more appropriate to define the sum of the DLPs of the acquisitions as DRL. For CT system 1 and 7 with the highest dose values for PRT, all acquisitions were performed with dose values that are commonly used for the examination of intervertebral discs with diagnostic image quality (e.g., CTDI_vol_ = 25 mGy for one acquisition, [32]). For the systems with low DLPs, only a few acquisitions were performed (approx. 3), and the CTDI_vol_ and DLP were a few 1mGy or 1mGy·cm, respectively, for the acquisitions. In consultation with the physicians performing the scans, these parameter values were sufficient to assess the positioning of the needle and the distribution of the drug. The sequential mode with a few rotations is generally associated with a lower DLP than an acquisition in helical mode.

As they are by far the most common acquisitions, four auxiliary series (no. 20–23, Table 1) were also included in the survey. Inter-system dose differences of up to a factor of 29 were found for the CTDI_vol_ from different systems. There were also dose differences of up to a factor of 10 between the same scanner types. Auxiliary series do not require diagnostic image quality, and dose-related parameter values can, therefore, be harmonized more easily than for other procedures.

Due to the participation of users from different countries, a more robust database could be obtained in this survey. As initially assumed, there are no significant differences in CT dose values between different countries in (central) Europe. This result should be an incentive for national authorities of different countries to cooperate more closely with each other, for instance, to define common DRLs.

This survey has some limitations. First, the participants were asked to report the quartiles from at least 10 representative DLP and CTDI_vol_ values. This corresponds to the German and European method to determine DRLs [14, 33]. It contradicts the ICRP approach [5], recommending a database of at least 20 dose values. However, 90% of all dose data collected in this survey was based on more than 20 dose values for a reference procedure. Second, the weight or body mass index of patients was not documented. Due to the large number of dose data (in some cases several hundred values), we assume, however, a weight distribution that corresponds to the reference person (of the respective procedure). Third, pediatric patient data were not included. Fourth, the image quality achieved was not evaluated. Therefore, there is the risk that dose values were not adapted to the required image quality of the specific clinical task. Lastly, the DRLs derived in this study do not necessarily represent the exposure practice in the respective countries, and a bias could arise from the fact that more large facilities with modern equipment and available medical physicists responded than the average.

## Conclusion

In this international multicenter survey, dose values from 87 CT systems from Austria, Germany, Italy, and Switzerland were collected to define clinical DRLs for 23 CT procedures for which national DRLs have not been specified yet. Dose values varied substantially between systems, even though all facilities were supervised by experienced medical physicists. The clinical DRLs obtained in this survey can be the basis for users, medical physicists, external auditors or authorities to evaluate and optimize local exposure practices ([5, 33]) or to establish new national DRLs. This survey underlines the importance of international cooperation between users of ionizing radiation to share knowledge and experience, to create a larger database for setting new DRLs and to harmonize exposure practices across national borders.

## Data Availability

The processed output (mean and quartiles of dose indices such as the DLP or CTDI_vol_) of the dose management systems of the (co-) authors was used to obtain the results reported in the article. All the outputs were received and analyzed by the authors K. Jungnickel, G. Stamm and A. Schegerer.

## Abbreviations

AP: Anterior-posterior
CAP: Clinical task, anatomical region and procedural technique
CCTA: Coronary CTA
CT: Computed tomography
CTA: CT angiography
CTDI_vol_: Pitch-corrected CT dose index
CTP: Cerebral CT perfusion
DLP: Dose length product
DMS: Dose management system
DRL: Diagnostic reference level
HPH: (Prospectively ECG-triggered) high-pitch helical
ICRP: International Commission on Radiological Protection
LAT: Lateral
PA: Posterior-anterior
preTAVI/R: CT examination before transcatheter aortic valve implantation/replacement
proECG: Prospectively ECG-triggered sequential mode
PRT: CT-guided periradicular therapy
retroECG: Retrospectively ECG-gated mode
U: Tube voltage

## References

[1] International Atomic Energy Agency (2014) Radiation protection and safety of radiation sources: international basic safety standards. General safety requirements Part 3. STI/PUB/1578. 10.61092/iaea.u2pu-60vm

[2] International Commission on Radiological Protection (2007) The 2007 recommendations of the ICRP. ICRP Publication 103. Ann ICRP 37:1–332 10.1016/j.icrp.2007.10.003 18082557

[3] Federal Assembly of the Swiss Confederation (1991) Radiological Protection Act. SR 814.50. www.fedlex.admin.ch/eli/cc/1994/1933_1933_1933/de. Accessed February 24, 2026

[4] German Federal Cabinet (2018) Ordinance on protection against the harmful effects of ionising radiation. Bundesgesetzblatt I:2034–2208

[5] International Commission on Radiological Protection (2017) Diagnostic reference levels in medical imaging. ICRP Publication 135. Ann ICRP 46:1–144 10.1177/0146645317717209 29065694

[6] European Commission (2014) Radiation protection 180: diagnostic reference levels in thirty-six European countries. Radiation Protection 180 Part 2/2. Publications Office of the European Union, Luxembourg

[7] European Commission, Jaschke W, Clark J et al (2021) European study on clinical diagnostic reference levels for X-ray medical imaging: EUCLID. Radiation Protection 195. Publications Office of the European Union, Luxembourg

[8] Paulo G, Damilakis J, Tsapaki V et al (2020) Diagnostic reference levels based on clinical indications in computed tomography: a literature review. Insights Imaging 11:96 32804275 10.1186/s13244-020-00899-y PMC7431477

[9] Ruiz-Cruces R, Vano E, Carrera-Magariño F et al (2016) Diagnostic reference levels and complexity indices in interventional radiology: a national programme. Eur Radiol 26:4268–4276 27384609 10.1007/s00330-016-4334-2

[10] Schegerer AA, Frija G, Paulo G et al (2021) Radiation dose and diagnostic reference levels for four interventional radiology procedures: results of the prospective European multicenter survey EUCLID. Eur Radiol 31:9346–9360 33991223 10.1007/s00330-021-08029-y

[11] Almén A, Guðjónsdóttir J, Heimland N, Højgaard B, Waltenburg H, Widmark A (2022) Paediatric diagnostic reference levels for common radiological examinations using the European guidelines. Br J Radiol 95:20210700 34898256 10.1259/bjr.20210700 PMC8822550

[12] Schegerer AA, Stamm G, Aberle C et al (2025) International survey on diagnostic reference levels based on clinical indications in plain radiography. Eur Radiol 35:3336–3346 10.1007/s00330-024-11224-2 PMC12081480 39630193

[13] Widmark A (2018) Diagnostic reference level (DRL) in Norway 2017. Results, revision and establishment of new DRL. Report of the Norwegian Radiation Protection Authority (NRPA), 3. 10.13140/RG.2.2.29964.21120

[14] Schegerer AA, Nagel HD, Stamm G, Adam G, Brix G (2017) Current CT practice in Germany: results and implications of a nationwide survey. Eur J Radiol 90:114–128 28583622 10.1016/j.ejrad.2017.02.021

[15] Dupont L, Aberle C, Botsikas D et al (2023) Proposed DRLs for mammography in Switzerland. J Radiol Prot. 10.1088/1361-6498/ad1037c1088 10.1088/1361-6498/ad37c8 38530290

[16] Schegerer A, Loose R, Heuser LJ, Brix G (2019) Diagnostic reference levels for diagnostic and interventional X-Ray procedures in Germany: update and handling. Rofo 191:739–751 30665250 10.1055/a-0824-7603

[17] Samara ET, Fitousi N, Bosmans H (2022) Quality assurance of dose management systems. Phys Med 99:10–15 35598480 10.1016/j.ejmp.2022.05.002

[18] International Electrotechnical Commission (2019) IEC 61223: Evaluation and routine testing in medical imaging departments—Part 3–5: Acceptance tests and constancy tests—imaging performance of computed tomography X-ray equipment (IEC 61223-3-5:2019 + Cor. 1:2022); IEC 61223-3-5

[19] Bundesärztekammer (2023) Guidelines on quality assurance in computed tomography (Leitlinie der Bundesärztekammer zur Qualitätssicherung in der Computertomographie). Deutsches Ärzteblatt. 10.3238/arztebl.2023.LL_Qualitaetssicherung_Computertomographie

[20] Zensen S, Guberina N, Opitz M et al (2021) Radiation exposure of computed tomography imaging for the assessment of acute stroke. Neuroradiology 63:511–518 32901338 10.1007/s00234-020-02548-z PMC7966220

[21] Cros M, Geleijns J, Joemai RM, Salvadó M (2016) Perfusion CT of the brain and liver and of lung tumors: use of Monte Carlo simulation for patient dose estimation for examinations with a cone-beam 320-MDCT scanner. AJR Am J Roentgenol 206:129–135 26700344 10.2214/AJR.15.14913

[22] Kasasbeh AS, Christensen S, Straka M et al (2016) Optimal computed tomographic perfusion scan duration for assessment of acute stroke lesion volumes. Stroke 47:2966–2971 27895299 10.1161/STROKEAHA.116.014177 PMC5134896

[23] Ramirez-Giraldo JC, Thompson SM, Krishnamurthi G et al (2013) Evaluation of strategies to reduce radiation dose in perfusion CT imaging using a reproducible biologic phantom. AJR Am J Roentgenol 200:W621–W627 23701093 10.2214/AJR.12.9413

[24] Kollefrath A, Stamm G, Schegerer A, Ammon J, Jungnickel K (2021) Optimization in CT imaging. In: Georg D, Birkfelnner W (eds) Joint Conference of the ÖGMP, DGMP and SGSMP

[25] International Commission on Radiological Protection (2013) Radiological protection in cardiology. ICRP Publication 120. Ann ICRP 42:1–125 10.1016/j.icrp.2012.09.001 23141687

[26] Kosmala A, Petritsch B, Weng AM, Bley TA, Gassenmaier T (2019) Radiation dose of coronary CT angiography with a third-generation dual-source CT in a “real-world” patient population. Eur Radiol 29:4341–4348 30506216 10.1007/s00330-018-5856-6

[27] Yang CC, Law WY, Lu KM, Wu TH (2019) Relationship between heart rate and optimal reconstruction phase in coronary CT angiography performed on a 256-slice multidetector CT. Br J Radiol 92:20180945 31322906 10.1259/bjr.20180945 PMC6732907

[28] Maroules CD, Rybicki FJ, Ghoshhajra BB et al (2023) 2022 Use of coronary computed tomographic angiography for patients presenting with acute chest pain to the emergency department: an expert consensus document of the Society of Cardiovascular Computed Tomography (SCCT): endorsed by the American College of Radiology (ACR) and North American Society for Cardiovascular Imaging (NASCI). J Cardiovasc Comput Tomogr 17:146–163 36253281 10.1016/j.jcct.2022.09.003

[29] Hedgire SS, Baliyan V, Ghoshhajra BB, Kalra MK (2017) Recent advances in cardiac computed tomography dose reduction strategies: a review of scientific evidence and technical developments. J Med Imaging 4:31211 10.1117/1.JMI.4.3.031211 PMC5571428 28894760

[30] Kędzierski B, Macek P, Dziadkowiec-Macek B, Truszkiewicz K, Poreba R, Gac P (2023) Radiation doses in cardiovascular computed tomography. Life 13:990 37109519 10.3390/life13040990 PMC10141413

[31] Shnayien S, Beetz NL, Bressem KK, Hamm B, Niehues SM (2023) Comparison of a high-pitch non-ECG-gated and a prospective ECG-gated protocol for preprocedural computed tomography imaging before TAVI/TAVR. Rofo 195:139–147 36063835 10.1055/a-1898-6504

[32] Bundesamt für Gesundheit (2018) Diagnostische referenzwerte in der computertomographie. Wegleitung R-06-06, 2. Revision vom 15.06.2018. www.bag.admin.ch/dam/de/sd-web/o9TMt9dOFYDC/R-06-06wd.pdf. Accessed February 24, 2026

[33] European Society of Radiology (2025) Esperanto—ESR guide to clinical audit in radiology, 4th edition. www.myesr.org/quality-safety/clinical-audit/. Accessed February 24, 2026

